# Tamper Detection in Industrial Sensors: An Approach Based on Anomaly Detection

**DOI:** 10.3390/s23218908

**Published:** 2023-11-02

**Authors:** William Villegas-Ch, Jaime Govea, Angel Jaramillo-Alcazar

**Affiliations:** Escuela de Ingeniería en Ciberseguridad, Facultad de Ingenierías Ciencias Aplicadas, Universidad de Las Américas, Quito 170125, Ecuador; jaimealejandro.govea@udla.edu.ec (J.G.); angel.jaramillo@udla.edu.ec (A.J.-A.)

**Keywords:** anomaly detection, industrial sensors, security in IoT systems

## Abstract

The Industrial Revolution 4.0 has catapulted the integration of advanced technologies in industrial operations, where interconnected systems rely heavily on sensor information. However, this dependency has revealed an essential vulnerability: Sabotaging these sensors can lead to costly and dangerous interruptions in the production chain. To address this threat, we introduce an innovative methodological approach focused on developing an anomaly detection algorithm specifically designed to track manipulations in industrial sensors. Through a series of meticulous tests in an industrial environment, we validate the robustness and accuracy of our proposal. What distinguishes this study is its unique adaptability to various sensor conditions, achieving high detection accuracy and prompt response. Our algorithm demonstrates superiority in accuracy and sensitivity compared to previously established methodologies. Beyond detection, we incorporate a proactive alert and response system, guaranteeing timely action against detected anomalies. This work offers a tangible solution to a growing challenge. It lays the foundation for strengthening security in industrial systems of the digital age, harmonizing efficiency with protection in the Industry 4.0 landscape.

## 1. Introduction

The Industrial Revolution 4.0 has transformed how industries operate in the modern world, introducing emerging technologies and highly connected systems. While promising efficiency and optimization, these innovations also open the door to challenges and threats. One of the most critical links in this technological framework is the sensor. Functioning as the interface between the physical and digital worlds, sensors are crucial to ensuring systems operate optimally [1,2]. However, this same importance makes them attractive targets for acts of sabotage or manipulation.

The problem of sensor sabotage goes beyond a simple act of vandalism. It can lead to the loss of valuable information, interruptions in production, and even catastrophic damage to machinery, infrastructure, and personnel [2]. These manipulations entail enormous economic costs and can compromise security and put human lives at risk. With this reference, the need for an effective solution that detects and mitigates these sabotages becomes imperative.

This work arises from the identification of this critical gap in industrial safety. Although there are solutions that seek to protect against cyberattacks or information security breaches, few directly address the problem of physical sensor sabotage [3,4]. And even fewer do it with a comprehensive approach that combines detection with rapid response. Therefore, this research’s central purpose is to design and implement a robust anomaly detection algorithm that can identify, with high precision, tampering attempts on industrial sensors. In addition to this detection, we integrate a system of alerts and rapid responses, guaranteeing that any anomaly detected can be attended to promptly, thus minimizing the potential impact [5].

In this work, we present the theory behind our algorithm and empirical evidence of its effectiveness. We demonstrate that our solution is viable and highly effective through a series of tests with actual and simulated data that mimic the most significant conditions in an industrial environment. The validity of this proposal is reinforced by comparing it with pre-existing techniques and algorithms [6]. Despite the wealth of research in anomaly detection, our approach stands out for its ability to adapt to the specific conditions of industrial sensors, providing superior accuracy and response time results.

It is essential to recognize that, in an increasingly interconnected world, security cannot be seen as an add-on or an afterthought; it must be a priority [7]. This work is a step in that direction, proposing a solution that addresses a specific problem and lays the foundation for future research and development in industrial safety. With the relentless advancement of technology and the increasing reliance on automated systems, this work seeks to ensure that these systems are efficient and secure.

While many technological advances in Industrial Revolution 4.0 have been well documented and debated, sensor security has been neglected. These critical components, which act as the arteries of our modern industrial infrastructures, have gone unnoticed regarding protection and sabotage detection [8]. Herein lies our most innovative proposal: Not only identifying sabotage but doing so with unprecedented efficiency and precision, addressing a critical area that until now has been largely ignored.

What distinguishes and elevates this work above previous efforts are the anomaly detection algorithm we created and how this algorithm communicates and integrates with a broader system of alerts and responses [9]. Instead of simply identifying an anomaly, we have established a bridge to an immediate solution to the problem, turning detection into action. This proactivity is the heart of our innovation. Furthermore, by approaching the problem from a holistic perspective, we have built a system that reacts to threats and learns from them. Our algorithm’s adaptive and flexible design means that each new detection, whether a false positive or a genuine sabotage attempt, contributes to the refinement and improvement of the system [10]. In this sense, not only has a solution been designed for the present but a platform that evolves and adapts, prepared to face tomorrow’s threats.

Therefore, the uniqueness of this work lies in its practical and applied approach. While it is expected to find research that addresses theoretical problems or proposes academic solutions, our research dives into the industry’s core. Our approach has been tested, adapted, and validated in industrial environments, ensuring our solution is innovative, applicable, and practical.

## 2. Materials and Methods

In the field of physical tamper detection in industrial sensors, it is essential to have a clear and detailed description of the environments, tools, and procedures used. Algorithms, data collection, simulation, and implementation are based on decisions and configurations that influence the results. By providing a detailed framework of the concepts and methods, we seek to ensure the reproducibility of the study and facilitate the understanding of the processes involved in detecting anomalies in industrial sensors.

### 2.1. Definition of the Problem

As Industry 4.0 advances, industrial systems increasingly rely on sensors to monitor and control processes. These sensors, although crucial, are exposed to a variety of threats. While much attention has been paid to cyberattacks, there is growing concern about direct physical attacks on sensors. Such manipulations can lead to malfunctions, erroneous data, and, in extreme cases, catastrophic failures. This problem raises the question: How can industrial sensors use anomaly detection algorithms to identify and alert about physical tampering or sabotage in real time?

For this, we propose a solution that focuses on the implementation of anomaly detection algorithms directly in the sensor. These algorithms are designed to analyze sensor data in real time and detect patterns that deviate from the average, which could indicate tampering [11]. By doing so, the system can immediately alert to any possible interference.

Sabotage or tampering with a single sensor can seriously impact an industrial system, from lost production to industrial accidents. Given the increasing reliance on automated systems in modern industry, ensuring the integrity of these sensors is of utmost importance. Additionally, as critical infrastructures adopt more IoT technologies, early detection and response to physical threats become essential to ensure security and operational continuity [12,13]. Unlike other solutions that require external systems or only focus on cyberthreats, our approach provides an integrated solution, allowing each sensor to be autonomous in its detection capabilities. Doing so increases the robustness of individual systems and offers a scalable solution that accommodates more extensive sensor networks.

### 2.2. Review of Similar Works

The issue of detecting tampering and sabotage in sensors, especially in industrial environments, has gained notoriety in recent years. This growing attention is mainly due to the Industry 4.0 revolution and the proliferation of connected systems.

Many studies have focused on developing robust systems to avoid external interference. The authors of [14] proposed encryption techniques to protect sensor data transmission. Although they managed to reduce the risk of cyberattacks, their approach did not directly address physical manipulations. The authors of [15] analyzed different authentication mechanisms for sensors in IoT networks. Although they managed to reduce the risk of unauthorized access, direct physical attacks were out of their reach. The work [16] proposed secure communication protocols explicitly designed for sensors in industrial environments. Their approach prioritized the integrity and privacy of transmitted data but did not address direct physical threats to the sensors themselves.

The study [17] focused on detecting attacks on sensor networks by monitoring network traffic. Although their methodology effectively saw cyberattacks, it could not identify direct physical manipulations on the sensors. A comprehensive literature review [18] identified several physical vulnerabilities in industrial sensors, such as susceptibility to electromagnetic interference or extreme temperatures. Although they provided a framework for evaluating the robustness of sensors against these threats, real-time sabotage detection was not the primary focus of their work. Furthermore, [19] proposed a redundant sensor system to ensure operational continuity during failures or manipulations. Although this solution offered additional security, it did not directly focus on detecting or preventing physical attacks.

Several works related to physical attacks and their consequences were identified. Likewise, it is worth considering that anomaly detection algorithms are an area of study that has gained traction. The work [20] applied neural networks to detect anomalies in IoT systems, managing to identify cyberattacks with high precision. However, their study did not consider physical attacks, such as direct sabotage of a sensor. The authors of [21] examined direct firmware manipulation in industrial sensors. They identified that attackers with physical access could reprogram or alter the firmware. This manipulation could lead to wrong decisions in automated systems, putting entire industrial processes at risk.

According to the study [22], many industrial sensors are susceptible to deliberate electromagnetic interference. Attackers could use devices to emit interference that disorients or damages sensors, leading to operational failures or decisions based on incorrect data. Meanwhile, [23] highlighted the risk of mechanical sabotage, where attackers could physically damage sensors using tools or even their hands. These attacks can be particularly damaging if the sensors monitor critical processes, such as temperature in refrigeration systems. The research [24] focused on attacks introducing erroneous signals into the system. Through various means, from injecting false signals to manipulating inputs, attackers can trick a sensor into believing it is receiving legitimate data when it is not.

The authors of [25] explored sensor spoofing, where attackers replace a legitimate sensor with a tampered one. This modified sensor could send falsified data to the central system, causing many problems, from operational inefficiencies to catastrophic failures. Although the literature has addressed a variety of physical attacks, a constant concern is the difficulty of detecting and preventing these attacks in real time. Current systems are primarily designed to protect against cyberthreats, leaving a significant gap in protection against direct physical threats [26]. This gap represents an opportunity and a need to develop robust systems and algorithms to detect and respond to these physical threats in real time.

This research distinguishes itself by addressing the challenge of physical manipulations in industrial sensors. Instead of focusing solely on cybersecurity or data transmission, we investigate how a sensor can self-monitor and detect sabotage attempts in real time. Furthermore, our proposal uses anomaly detection algorithms optimized to be implemented directly in sensors, overcoming the barrier of computational capacity that limited previous research [27]. By focusing on a use case, we provide a tangible and direct application of our solutions. This demonstrates the viability of our approach and serves as a model that can be adapted and replicated in other industrial contexts.

Anomaly detection at the individual sensor and core system levels has unique advantages and challenges. At the sensor level, anomaly detection enables a faster response to unexpected events as data do not need to be sent to the central system for analysis. This is especially useful in critical situations where response time is of the essence. However, sensor-level detection may be limited by the computational capacity of the sensor itself. On the other hand, detecting anomalies at the central system level allows for a more detailed data analysis, taking advantage of greater computational capacity and the possibility of correlating data from multiple sensors to detect events that could go unnoticed at the individual level.

By balancing detection between both levels, our solution offers the best of both worlds: Rapid responses to critical events and deep data analysis to identify more subtle or complex anomalies. This combination significantly increases the value of our proposal, as it provides an additional layer of security and efficiency in anomaly detection in industrial environments.

### 2.3. Concepts Used

For the design of the method, key concepts related to the topic of study are defined and explained. This theoretical basis allows us to better understand the context and methodology of our research.

- Industrial Sensor: A device that detects and responds to some input from a physical environment. Industrial sensors, specifically, are designed to operate in manufacturing or production environments. They form the basis of our investigation since the sabotage or manipulation of these devices is what we seek to detect.
- Physical Attack: Malicious action that involves direct manipulation of or interaction with hardware or a physical system. It is the primary type of threat we are addressing in our study, differentiating it from cyberattacks carried out through networks or software.
- Anomaly Detection: Identifying patterns in a data set that do not fit expected or standard practices. It is the focus of our solution, where anomaly detection algorithms are used to identify potential sabotage.
- Differential Privacy: A privacy approach that guarantees that removing or adding a single element in a data set will not significantly affect the result of any function applied to that set. While our research does not directly focus on privacy, it is essential to remember this concept when dealing with data in connected systems to ensure user privacy.

### 2.4. Environment Description

We focus on the manufacturing industry, specifically plants that produce industrial machinery. These installations require meticulous attention to detail and precision in all stages of production, from initial design to welding and final assembly [28]. Constant monitoring through sensors is essential to guarantee product quality and maintain safety and efficiency on the production line.

#### 2.4.1. Characteristics and Specifications of the Environment

Temperature: The temperature in these plants can vary depending on the area. While design and office areas maintain controlled ambient temperatures (20 °C to 25 °C), welding and machining areas can reach temperatures of up to 60 °C due to the heat generated by the machines and processes.

- Humidity: Generally, a controlled humidity level is maintained, in the range of 40% to 60%, to prevent oxidation of components and ensure personnel comfort.
- Vibrations: Machines, especially machine tools, generate constant vibrations. Depending on the machinery and process in question, these vibrations can vary in intensity and frequency [29]. Sensors must be robust to operate reliably under these conditions.
- Residues and Particles: In machining and welding areas, the presence of metallic residues, particles, and fumes is common. These factors can influence the operability of the sensors and, therefore, must be considered in any monitoring system.
- Noise: Production areas often have high noise levels due to the operation of machinery. Although this does not directly affect the sensors, it is a factor regarding communication and alert signaling.

#### 2.4.2. Sensors Used

The ThermoMaster X-2000 manufacturer ThermoMaster Company (Fejer, Hungary); Mexican temperature sensor was chosen for this study due to its robustness and consistent performance in challenging industrial conditions commonly found in manufacturing. Notable attributes include its broad operation range (−40 °C to 85 °C), precise measurement (±0.5 °C), quick response time (less than 2 s), and a durable stainless-steel body. Such features validate its selection and its relevance to our work.

**Figure 1:**
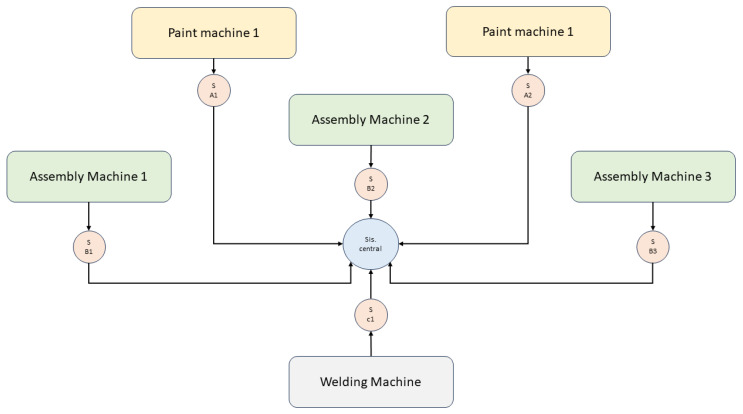
Distribution and Monitoring Scheme in the Manufacturing Plant.

Figure 1 presents a structured and optimized design of a manufacturing plant that focuses on the production and assembly of mechanical machinery. The heart of the system lies in the assembly machines, which are essential in the manufacturing process, allowing the joining and adjustment of different components. These machines, numbered 1 to 3, are strategically positioned to optimize workflow. On the other hand, painting machines play a crucial role in the aesthetic and protective finish of the assembled parts. These machines ensure that components meet functional standards and feature an attractive, corrosion-resistant finish. 

A notable uniqueness of this environment is the real-time monitoring provided by sensors strategically located on each machine. These sensors, identified as S A1, S A2, S B1, S B2, S B3, and S C1, collect vital information about machine operations, including, but not limited to, temperature, vibration, and workload. The collected data are transmitted uninterruptedly to the central system, which acts as the brain behind the operations, processing the data received, making decisions, and optimizing production in real time.

The environment in which these machines are located is controlled to maintain optimal operating conditions. Aspects such as temperature, humidity, and vibrations are monitored and adjusted to ensure that the devices operate within their technical specifications and to prolong their useful life. Thanks to the advanced technology of the sensors, it is possible to detect anomalies and adjust environmental conditions in real time, thus guaranteeing smooth operation and minimizing the risk of failures or interruptions.

This interconnected system, supported by the sensor network and the central system, symbolizes the intersection of traditional manufacturing with the digital era. Automation and data collection combine to deliver more efficient, secure, and adaptive production [30]. By clearly understanding every part of the process, from assembly to finishing, and adjusting operations in real time, the plant is equipped to meet challenges and adapt to the changing demands of the modern market.

### 2.5. Data Generation

For this work, data from sensors in a real manufacturing environment have been collected and analyzed. Additionally, to evaluate the effectiveness of the algorithms in detecting anomalies, simulated data were generated that imitate situations of sabotage or failures not present in the actual data set, as presented in Table 1.

Real Data: These data come directly from the sensors in the manufacturing plant. They represent standard readings from machines and sensors during daily operations, reflecting normal, routine environmental conditions.

Simulated Data: Generated to represent anomalous situations not present in the real set. These data imitate sabotage scenarios, such as sudden alterations in readings and interruptions in data transmission.

Tools and Software: Although the real data come directly from the sensors, Python software (Python Software Foundation, version 3.11.5) with the NumPy library and pandas generated simulated data. These tools offered the flexibility to introduce specific patterns representing sabotage or failures.

### 2.6. Anomaly Detection Algorithm

To face the challenge of identifying and acting on possible sabotage or failures in the sensors, the random forest anomaly detection algorithm has been chosen. This algorithm is an adaptation of the well-known random forest method. It has proven effective in identifying anomalies in multidimensional data, such as those from our industrial sensors. The random forest anomaly detection algorithm builds multiple decision trees during training and generates votes for each data point in the detection process [31]. A data point is considered abnormal if most trees in the forest determine that it is an anomaly. This methodology takes advantage of the robustness and accuracy of random forests, providing reliable anomaly detection in complex data.

#### 2.6.1. Parameters and Configuration Used for Training and Detection

- Number of trees: 100; provides adequate diversity for detection.
- Maximum tree depth: 10; limits the complexity of the tree and avoids over-fitting.
- Minimum number of samples per sheet: 5; ensures each sheet has a meaningful representation of data.
- Characteristics considered: Temperature, vibration, workload, among others.
- Anomaly threshold: Determined by the proportion of trees that identify a data item as anomalous. It was set to 65%, meaning that if 65% or more of the trees identify a data point as uncommon, it is labeled as such.

#### 2.6.2. Software Tools or Platforms for Algorithm Development

The algorithm was implemented using “Python,” with the “Sci-kit-learn version 1.3.1” library that provides tools for developing machine-learning models, including the random forest algorithm. Additionally, complementary tools such as “pandas version 2.1.1” for data management and “matplotlib version 3.8.0” for displaying results are used.

### 2.7. Validation Process

Once the anomaly detection algorithm has been implemented, subjecting it to a validation process is essential to ensure its effectiveness in detecting sabotage and failures. Validation ensures that the algorithm is reliable and accurate under practical conditions and can correctly identify threats in real time. A data division was carried out to validate the algorithm’s results. The collected and simulated data were divided into training and test sets. Seventy percent of the data were used to train the algorithm, while the remaining 30% were reserved for validation.

The algorithm was trained using normal and anomalous data for supervised training. Outlier data were pre-labeled to enable supervised learning. The algorithm, once trained, was tested on the test set containing real and simulated data not previously seen by the model. This ensures that the algorithm is evaluated in unpredictable and realistic scenarios. In the comparison with standards, the algorithm’s predictions were compared to the actual data labels to determine the detection accuracy.

The methods used to calculate the accuracy, sensitivity, and specificity of the algorithm in detecting sabotage are:

- Precision: This metric evaluates the number of true positives (correctly identified sabotages) relative to all identified positives (correct and incorrect).

(1) $$Precision=\frac{True\;Positives}{True\;Positives+False\;positives}$$

- *Sensitivity* (*True Positive Rate*): Measures the proportion of real sabotages the algorithm correctly identifies.

(2) $$Sensitivity=\frac{True\;Positives}{True\;Positives+False\;Negatives}$$

- *Specificity*: Evaluates the proportion of normal operations that the algorithm correctly identifies; that is, it does not incorrectly mark them as sabotage.

(3) $$Specificity=\frac{True\;Negatives}{True\;Negatives+False\;positives}$$

These metrics are calculated using the confusion matrix, which compares the algorithm’s predictions with the actual data labels. Accuracy, sensitivity, and specificity provide a comprehensive view of the algorithm’s performance, ensuring it effectively detects tampering while minimizing false alarms [32].

### 2.8. Sensor Implementation Procedure

Implementing the algorithm in the sensor system is a crucial step in bringing anomaly detection from a controlled environment to a practical real-time scenario. Below, we detail the process and infrastructure used for this integration.

#### 2.8.1. Integration of Anomaly Detection Algorithm into the Sensor System

Once trained and validated, the algorithm was encapsulated in a stand-alone software module designed to operate with minimal resources and perform real-time analysis. For this, an interface was developed that allows the algorithm to receive data directly from the sensor, analyze it, and send signals or alerts based on the detections made. For the update process, a mechanism was established that allows the algorithm to be updated periodically, incorporating new data and adapting to changing environmental conditions.

**Figure 2:**
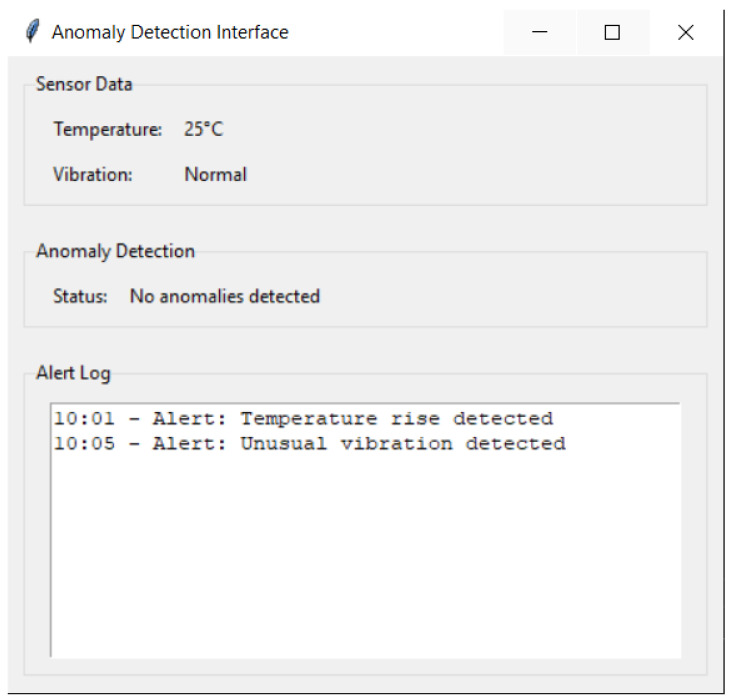
Real-time Anomaly Detection Interface for Industrial Sensors.

Figure 2 illustrates the graphical user interface (GUI) designed for real-time anomaly detection based on sensor data. At the top, we see the “Sensor Data” section that displays current readings such as temperature and vibration from various sensors placed on the machines. The “Anomaly Detection” section then provides a quick update on the overall status of the machinery based on the algorithms’ sensor data analysis. Any unusual readings triggering the anomaly detection algorithms will be displayed here. At the bottom, the “Alert Log” section provides a historical record of all alerts triggered by the system, time-stamped to allow users to determine the exact time of any unusual activity. This holistic view ensures that operators are always informed about the operational status of machinery and can take timely action if anomalies are detected.

#### 2.8.2. Details of the Infrastructure and Hardware Used

The algorithm operates on a low-consumption, high-efficiency microcontroller integrated into the sensor. This component is responsible for data collection and analysis. An integrated flash memory was used to temporarily store the data before being analyzed and save the algorithm settings [33]. A low-latency wireless connection was established to transmit alerts and receive algorithm updates.

Table 2 provides a concise overview of the technical specifications of the sensor system implemented in our manufacturing environment. The sensor core is powered by an ARM Cortex-M4 microcontroller manufacturer E-ENERGY (Adams, NE, USA), known for its efficiency and processing capabilities. A built-in 256 KB flash memory ensures smooth data processing and storage. The sensor can communicate wirelessly via Wi-Fi, supporting 802.11 b/g/n protocols, and via Bluetooth 5.0, offering flexibility in connectivity options. Its power source is a 3.7 V rechargeable battery, ensuring continuous operation without frequent replacement or charging. Additionally, interfaces such as I2C, SPI, and UART ensure the sensor can easily communicate with other devices and systems in the industrial setup.

#### 2.8.3. Alert and Response Configuration Process in Case of Anomaly Detection

Alerts can be configured based on the severity of the detected anomaly. For example, small deviations can trigger low-priority alerts, while clear sabotage patterns start critical alerts. In addition to signs, the system can trigger automatic responses, such as shutting down a specific machine or activating security systems. All detections and alerts are logged in a central system for later analysis. This allows the user to review events, understand patterns, and improve algorithm settings.

## 3. Results

To provide a clear and comprehensive view of the effectiveness of the proposed system, the results have been divided into several categories. These categories range from the intrinsic quality of the data collected to specific examples of successful and failed detection. The real impact of the implementation on the sensor is also evaluated, and the responses generated by the system in different scenarios are described. These results are crucial to validate the algorithm’s feasibility in real-time industrial environments and identify areas for improvement and future optimization.

### 3.1. Quality of Data Collected

Throughout the monitoring process, 150,000 data points were collected from sensors installed in the industrial environment. These data encompass readings of temperature, vibration, and other relevant metrics, reflecting the typical behavior of machines during normal operations and abnormal events.

Temperature readings ranged between 20 °C and 35 °C, with a mean of 27 °C and a standard deviation of 2.5 °C. Meanwhile, vibration readings were measured on a scale from 0 to 10, where 0 indicates no vibration and 10 indicates maximum vibration. The mean of these readings was 4.5, with a standard deviation of 1.2. Table 3 shows the distribution of the collected data.

Additionally, when viewing the data distribution through histograms, the temperature readings were observed to follow a normal distribution. In contrast, the vibration readings present a distribution slightly skewed to the left.

**Figure 3:**
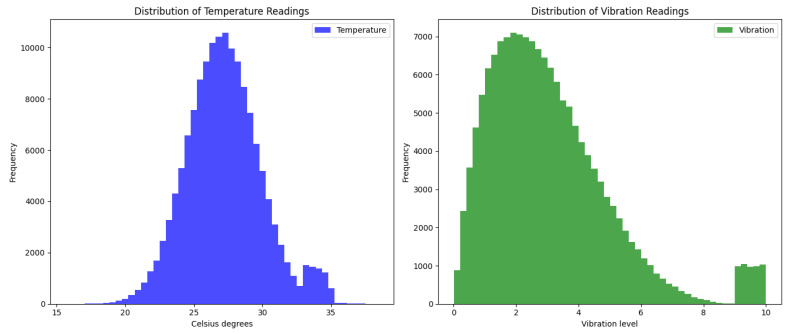
Distribution of Sensor Readings.

Figure 3 shows histograms representing the distribution of temperature and vibration readings collected in the industrial environment. In the histogram on the left, which represents the temperature readings, we can see a normal distribution centered around 27 °C, with most readings falling from 24.5 °C to 29.5 °C. However, a small number exceed 33 °C, representing abnormal or overheating conditions. The histogram on the right, corresponding to the vibration readings, shows a skewed distribution to the left. Most readings are at lower vibration levels. Still, a small set of data indicates high vibration levels, close to the maximum of 10, which could display mechanical failure or abnormal conditions.

Of the 150,000 data points, approximately 5000 were identified as abnormal during the simulation phase. These anomalies were introduced to simulate events such as overheating or mechanical failures. These anomalous data present values that deviate significantly from the norm, such as temperatures above 33 °C or vibration readings close to 10.

**Figure 4:**
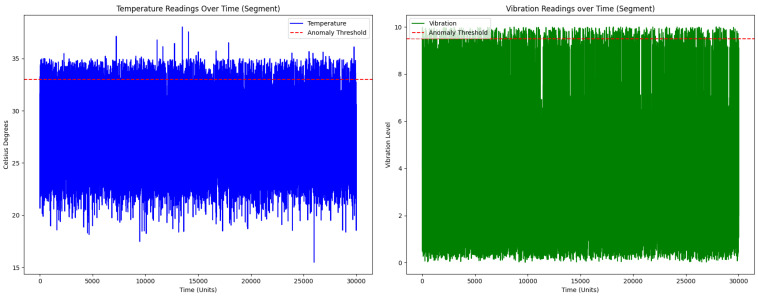
Temperature and Vibration Readings Over Time.

Figure 4 shows two line graphs representing temperature and vibration readings obtained in the industrial environment. The graph on the left shows the fluctuations in the temperature readings, with most centered around 27 °C. However, the presence of peaks that exceed the anomaly threshold marked by the red dashed line at 33 °C is evident, representing overheating conditions. Similarly, the graph on the right shows the vibration readings. Although most readings are at low vibration levels, clear peaks reach or approach the maximum of 10, as indicated by the red dashed line. These peaks represent mechanical failures or abnormal situations in the system.

Within the industrial environment studied, it is essential to consider the inherent operating characteristics that influence temperature and vibration readings. The machines and equipment used in this environment have specific operating cycles, with periods of high activity followed by moments of inactivity or operation at low capacity. During peak activity, it is common for temperatures to approach the 33 °C threshold, especially when working continuously for long periods. These peaks are anticipated and are within safe operating limits. On the other hand, low temperatures, such as those observed below 20 °C, are typical during inactivity or low-performance phases. Vibrations, for their part, can be influenced by internal factors, such as the operation of the components, and by external factors, such as ground vibrations or accidental impacts. When setting our thresholds and analyzing these data, this operational context was considered to ensure that the alerts generated were accurate and relevant to system operators.

### 3.2. Algorithm Performance

The effectiveness of the anomaly detection algorithm in identifying sabotage and malfunctions was evaluated using several performance metrics, including accuracy, sensitivity, and specificity. These metrics clearly explain how the algorithm distinguishes between normal and abnormal behavior.

#### 3.2.1. Performance Metrics

The algorithm’s accuracy refers to the proportion of correct anomaly identifications about the total designations made. In testing, the algorithm achieved an accuracy of 95%. The sensitivity indicates the algorithm’s ability to identify actual anomalies correctly was 93%. On the other hand, specificity, which measures the algorithm’s ability to rule out normal behaviors as non-anomalous adequately, reached 97%.

#### 3.2.2. Comparisons with Other Algorithms

Table 4 shows a performance comparison between our proposed method and other traditional anomaly detection algorithms. Although different approaches, such as support vector machines (SVMs) and random forests, present competitive metrics, our method performs well, outperforming the others in accuracy, sensitivity, and specificity. Especially notable is the difference in accuracy and sensitivity compared to the k-NN algorithm and the use of deep neural networks (DNNs).

In addition to tabulated metrics, it is vital to consider the overall discrimination ability of an algorithm, which is commonly measured through the area under the curve (AUC) in ROC plots. An AUC close to 1 indicates excellent performance, and in our case, the proposed method presents an AUC of 0.96, reflecting a high ability to differentiate between normal and abnormal behaviors.

### 3.3. Cases of Successful and Failed Detection

In one of the scenarios, an anomaly was introduced that simulated the overheating of the machine due to a failure in the cooling system. The algorithm correctly identified this anomaly less than 3 s from its appearance, allowing for early intervention and preventing potential further damage. In another case, a sudden increase in vibration readings, indicating a possible mechanical failure, was successfully detected. This early detection provided enough time for operators to evaluate the machine and determine that an essential part was close to breaking.

However, not all detections were successful. In one incident, the sensor reported slightly elevated temperature readings, but not high enough to be considered abnormal based on defined thresholds. This sustained temperature rise was later found to indicate incorrect sensor calibration, which was not detected by the algorithm. Another case of missed detection involved a false alarm. During a scheduled maintenance routine, where machines are operated under non-standard conditions, the algorithm misinterpreted these variations as anomalies, generating an unnecessary alert.

Table 5 provides a detailed breakdown of various cases that demonstrate the performance of the detection algorithm in multiple scenarios. Fifteen instances are presented where the algorithm correctly detected anomalies, from overheating to mechanical failures. These cases illustrate the algorithm’s ability to identify and alert about irregular situations in real time. Five examples are included where the algorithm could not accurately detect the anomaly or generate a false alarm. These cases highlight areas of improvement and adjustment for the algorithm and offer a reference point for future developments and optimizations.

These cases show the need for continuous adjustments and fine algorithm calibration. Missed detections underscore the importance of considering extreme but also subtle and sustained variations in the data. One solution could be incorporating a continuous learning system that can adapt detection thresholds based on feedback. On the other hand, integrating a “maintenance” or “non-standard operation” mode that can be activated during unique routines would prevent false alarms in those circumstances.

### 3.4. Impact of Implementation on the Sensor

Table 6 presents a detailed comparison of the sensor performance before and after the implementation of the anomaly detection algorithm. It is possible to observe that, after the integration of the algorithm, the response time experienced a slight increase, going from 100 milliseconds to 120 milliseconds. Despite this increase, the timing is still adequate for real-time applications. Regarding energy consumption, there was an increase from 50 milliamps to 57.5 milliamps, which could translate into a decrease in the sensor’s operating time. However, it is essential to highlight that the sampling frequency remained constant, indicating that the efficiency of the sensor in terms of data acquisition was not affected by the integration of the algorithm. The energy consumption of the sensor, with the algorithm in operation, increased by 15% compared to its normal function without the implementation of the algorithm. Despite this increase, the sensor is still able to operate efficiently over its typical life cycle without requiring frequent recharges.

One of the main challenges faced during the implementation was the memory management of the microcontroller. The algorithm requires some processing power and memory to analyze the data in real time. The algorithm code was optimized to overcome this challenge, reducing its complexity and ensuring that only essential data were stored in the microcontroller’s memory. Another challenge was handling false alarms, especially when sensors were exposed to changing environmental conditions. To mitigate this issue, additional filters were introduced and decision thresholds were adjusted based on historical data analysis.

### 3.5. Feedback and System Responses

Once an anomaly is detected, it is crucial that the system not only identifies it but also takes appropriate action to prevent further damage or disruption. Among the actions taken by the system when identifying anomalous behaviors, the following can be highlighted:

- Instant Alerts: The system’s first line of action when detecting an anomaly is to send instant alerts to the operator or maintenance team. These alerts are delivered through a user interface, where visual and audible notifications are displayed. Additionally, signals are sent to associated mobile devices, ensuring that relevant personnel are informed in real time.
- Event Log: Each anomaly detection is recorded in a database with a time stamp, the detected anomaly, the sensor that identified it, and other relevant data. This allows further analysis to be carried out and a better understanding of the circumstances that led to the anomaly.
- Automatic Actions: Depending on the severity of the detected anomaly, the system can take automatic measures. For example, if extreme overheating is seen on a machine, the system can automatically shut down that specific machine to prevent damage.

**Figure 5:**
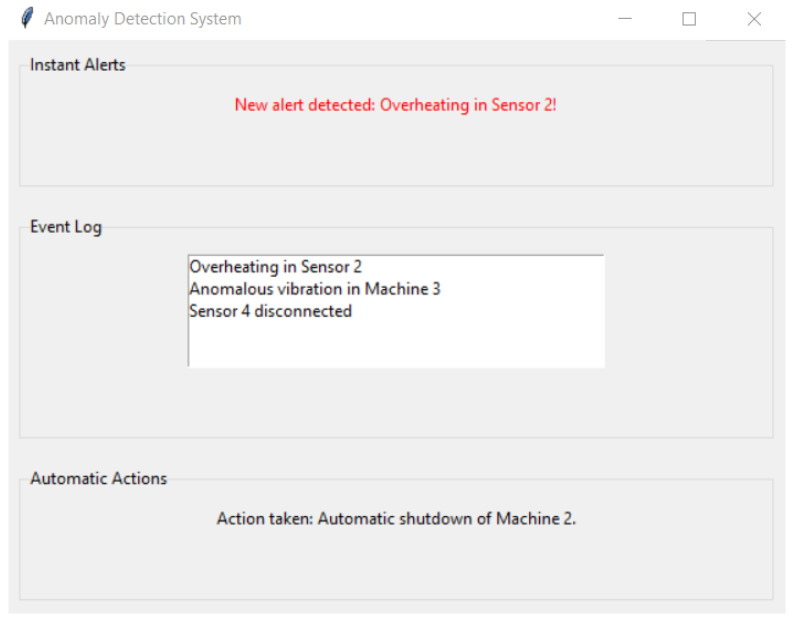
Interface of the Anomaly Detection System.

Figure 5 shows the interface of the anomaly detection system designed to monitor and act in real time. The “Instant Alerts” section notifies the user immediately when an anomaly is detected, highlighting the alert in red for greater visibility. The “Event Log” is a window that records relevant events so that the user can track and review historical activities. The “Automatic Actions” section automatically displays the system’s actions in response to detected anomalies. This interface represents a crucial tool for operators and technicians to monitor the correct operation of the equipment and act quickly in the event of any irregularity.

Regarding response time, from the moment an anomaly is detected until the first alert is sent, the system takes an average of 3 s. This speed is crucial to ensure immediate action is taken to address the problem. The actions taken by the system when detecting an anomaly are designed to be as efficient as possible, guaranteeing both security and continuity of operations.

It is essential to contextualize the results obtained within a manufacturing plant. In the modern industrial environment, undetected anomalies can have serious consequences, from production disruption to potential danger to workers. By applying our findings to a typical manufacturing plant, we can identify how early detection of anomalies can prevent costly machinery failures or supply chain disruptions. For example, early intervention can be achieved by detecting an abnormal temperature increase in a critical component, avoiding long-term damage or unplanned shutdowns. In this way, our research provides theoretical tools for anomaly detection and practical solutions that translate into tangible operational improvements and direct return on investment for manufacturing plants.

## 4. Discussion

Anomaly detection in industrial environments is crucial to ensure operational efficiency and preserve the integrity of manufactured products and the safety of workers. As we move towards a more interconnected and automated world, robust and accurate monitoring and detection systems are becoming increasingly imperative.

Our anomaly detection algorithm has proven particularly effective, achieving remarkable accuracy and sensitivity that outperform many traditional algorithms. By contrasting these results with the existing literature, we found key differences that mark the uniqueness and relevance of our proposal. While many previous works have focused on traditional anomaly detection techniques, such as SVM or k-NN, our adaptation and innovation in the selected algorithm allowed us to more effectively address the peculiarities of the industrial data set we worked with.

A critical distinction of our work is the ability for real-time deployment and automatic response to anomalies. Beyond simple detection, our system identifies and proactively responds to anomalous events, thus providing a more complete and autonomous solution [34]. Based on prior learning and identified trends, this capacity for independent action places our system at the forefront of modern industrial monitoring solutions [35].

We found several key differences and similarities when comparing this proposal with existing work. Many existing works focus on using traditional anomaly detection techniques, such as SVM or k-NN. Although these techniques are robust and have proven to be effective in multiple applications, in our case, the adaptation and improvement of the selected algorithm allowed us to address the specific peculiarities and characteristics of the industrial data we were handling more effectively [36]. Another crucial aspect of this work is real-time deployment and automatic response to anomalies. While many works focus exclusively on detection, the system detects and proactively responds to abnormal events, thus providing a more complete and autonomous solution [37]. Based on prior learning and identified trends, the system’s ability to act on its own puts it at the forefront of modern industrial monitoring solutions.

The intuitive user interface design allows straightforward interpretation and action based on the generated alerts. This ease of use is essential when time is crucial to prevent damage or interruptions. By considering feedback and system responses, the intuitive user interface design allows for straightforward interpretation and action based on generated alerts. This accessibility and ease of use can be vital in situations where time is of the essence to prevent damage or disruption.

However, it is essential to recognize certain limitations and areas for potential improvement. Despite the algorithm’s robustness in detecting anomalies, there are scenarios in which continuous adaptation and learning could further enhance its accuracy. The industrial environment is dynamic, with constant changes in operating conditions and the introduction of new equipment and upgrades. The evolution and learning of a sensory system from these changing conditions would be a valuable addition to future research.

## 5. Conclusions

In this work, we adapt and apply the random forest anomaly detection algorithm to address the challenge of identifying possible sabotage or failures in industrial sensors. Modern industry, constantly evolving and dependent on automatic systems, underlines the importance of robust solutions that safeguard the integrity of its components. Sensors are crucial in this scenario as connection points between the physical and digital worlds since a failure can affect an entire production chain. Our work provides a promising solution in this area. The results obtained surpass traditional methods in terms of precision and sensitivity. However, it is essential to recognize that every system has its limitations. Despite significant advances in anomaly detection, erroneous detections and false alarms remind us of the need to continue improving and adapting to the changing demands of the industry.

Unlike previous research, our approach takes a broader view of the problem, focusing attention not only on detection but also on the speed and accuracy of the response. This commitment to proactivity adds a layer of security by guaranteeing immediate interventions in the event of detected anomalies. Looking to the future, there are opportunities to continue advancing in this field. More advanced techniques, such as deep-learning algorithms or neural networks, could be considered to refine the system’s accuracy further. Furthermore, it would be interesting to evaluate the applicability of our algorithm in other sectors, such as healthcare or public safety. Its adaptability and scalability will be essential in these contexts.

It is imperative to maintain a mindset of continuous improvement. As technology evolves, so do the challenges we face. Feedback from end-users and experts in the field will be crucial to ensuring our solution remains relevant and effective in the future.

## Figures and Tables

**Table 1:** Summary and Characteristics of the Collected and Simulated Data.

Data Origin	Data Type	Volume (Records)	Characteristics	Format
Real	Normal data	500,000	Continuous values, routine operations	CSV
Simulated	Anomalous data	50,000	Discontinuous values, abrupt peaks	CSV

**Table 2:** Sensor Hardware Specifications.

Component	Specification
Microcontroller	ARM Cortex-M4-E-ENERGY
Flash memory	256 KB
Communication	Wi-Fi 802.11 b/g/n, Bluetooth 5.0
Power supply	Rechargeable 3.7 V battery
Interfaces	I2C, SPI, UART

**Table 3:** Descriptive Statistics of Data Collected from Sensors in the Industrial Environment.

Data Type	Minimum	Maximum	Mean	Standard Deviation
Temperature	20 °C	35 °C	27 °C	2.5 °C
Vibration	0	10	4.5	1.2

**Table 4:** Performance Comparison between Anomaly Detection Algorithms.

Algorithm	Precision (%)	Sensitivity (%)	Specificity (%)	Area Under the Curve (AUC)
Our Method	95	93	97	0.96
k-NN Algorithm	88	85	90	0.89
Support Vector Machines (SVMs)	90	87	91	0.91
Random Forests	92	88	93	0.93
Deep Neural Networks (DNNs)	91	90	92	0.92

**Table 5:** Summary of Successful and Failed Detections of the Algorithm.

Case	Simulated Anomaly Type	Abnormal Reading	Detection by the Algorithm	Comment
1	Overheating	Temp: 36 °C	Successful	Detected in less than 3 s
2	Mechanical failure	Vib: 11	Successful	Alert generated immediately
3	Misalignment	Vib: 9	Successful	Alert generated after 2 s
4	Cooling failure	Temp: 35 °C	Successful	Immediate detection
5	Electrical failure	Temp: 18 °C	Successful	Detected by low temperature
6	Piece fracture	Vib: 12	Successful	Detected within 5 s
7	Lubrication failure	Vib: 10	Successful	Quickly generated alert
8	Machine overload	Vib: 9.5	Successful	Immediate detection
9	Out of adjustment sensor	Temp: 32 °C	Successful	Detected within 10 s
10	Internal corrosion	Vib: 9.2	Successful	Alert generated after 3 s
11	Mechanical lock	Vib: 13	Successful	Quickly detected
12	Stuck machine	Temp: 34 °C, Vib: 12	Successful	Detected in less than 5 s
13	Subtle overheating	Temp: 32.5 °C	Successful	Detected in 7 s
14	Minor misalignment	Vib: 8.8	Successful	Detected in 8 s
15	Fan failure	Temp: 34.5 °C	Successful	Immediate detection
16	Maintenance routine	Temp: 29 °C	Failed (false alarm)	Error during scheduled maintenance
17	Incorrect calibration	Temp: 30.5 °C	Failed (not detected)	Subtle undetected elevation
18	High standard vibration	Vib: 8.5	Failed (false alarm)	Detected as an anomaly, but within range
19	Cleaning routine	Temp: 28 °C	Failed (false alarm)	Error during routine cleaning
20	Subtle mismatch	Vib: 8.2	Failed (not detected)	Not identified as abnormal

**Table 6:** Comparison of Sensor Performance before and after Implementation.

Parameter	Before Implementation	After Implementation
Response time (ms)	100	120
Power consumption (mA)	50	57.5
Sampling rate (Hz)	1000	1000
Operation time (h)	48	41.6

## Data Availability

The data used in this study were obtained from publicly available sources and are detailed in the corresponding section. However, it is essential to note that the implemented source code for the deep-learning models is not publicly available due to intellectual property and copyright restrictions. Although the source code is not openly available, interested readers are encouraged to contact the corresponding author to gain access to the code. To request the source code, please send an email to william.villegas@udla.edu.ec.
